# Navigating new horizons: Prospects of NET-targeted radiopharmaceuticals in precision medicine

**DOI:** 10.7150/thno.96743

**Published:** 2024-05-19

**Authors:** Takahiro Higuchi, Xinyu Chen, Rudolf A Werner

**Affiliations:** 1 Department of Nuclear Medicine and Comprehensive Heart Failure Center, University Hospital of Würzburg, Würzburg, Germany.; 2 Faculty of Medicine, Dentistry and Pharmaceutical Sciences, Okayama University, Okayama, Japan.; 3 Nuclear Medicine, Faculty of Medicine, University of Augsburg, Augsburg, Germany.; 4 DZHK (German Centre for Cardiovascular Research), Partner Site Frankfurt Rhine-Main, Frankfurt, Germany.; 5 Goethe University Frankfurt, Department of Nuclear Medicine, Clinic for Radiology and Nuclear Medicine, Frankfurt, Germany.; 6 German Cancer Consortium (DKTK), Partner Site Frankfurt/Mainz and German Cancer Research Center (DKFZ), Heidelberg, Germany.; 7 The Russell H Morgan Department of Radiology and Radiological Sciences, Division of Nuclear Medicine and Molecular Imaging, Johns Hopkins School of Medicine, Baltimore, MD, United States.

**Keywords:** Norepinephrine, PET, Astatine, MIBG, Alpha-particle, neuroendocrine tumor

## Abstract

In the evolving landscape of precision medicine, NET-targeted radiopharmaceuticals are emerging as pivotal tools for the diagnosis and treatment of a range of conditions, from heart failure and neurodegenerative disorders to neuroendocrine cancers. This review evaluates the advancements offered by ^18^F-labeled PET tracers and ^211^At alpha-particle therapy, juxtaposed with current ^123^I-MIBG SPECT and ^131^I-MIBG therapies. The enhanced spatial resolution and capability for quantitative analysis render ^18^F-labeled PET tracers potential candidates for improved detection and management of diseases. Alpha-particle therapy with ^211^At may offer increased specificity and tumoricidal efficacy, pointing towards a shift in therapeutic protocols. While preliminary data is promising, these innovative approaches require thorough validation against current modalities. Ongoing clinical trials are pivotal to confirm the expected clinical benefits and to address safety concerns. This review underscores the need for rigorous research to verify the clinical utility of NET-targeted radiopharmaceuticals, which may redefine precision medicine paradigms and significantly impact patient care.

## Introduction

*NET and its role in various diseases.* The Norepinephrine Transporter (NET) is a critical protein that regulates the concentration of norepinephrine in both the central and peripheral nervous systems by facilitating the reuptake of this neurotransmitter from the synaptic cleft. The functional integrity of NET is essential for the maintenance of normal catecholaminergic neurotransmission, influencing a host of physiological processes ranging from mood regulation 1, 2 to the control of blood pressure 1. Disruptions in NET function have been implicated in a diverse array of diseases. In cardiovascular medicine, NET abnormalities can lead to dysregulated sympathetic activity, contributing to the pathogenesis of conditions such as heart failure 3. In the realm of psychiatric and neurodegenerative disorders, variations in NET expression and function are associated with conditions such as depression 1, attention-deficit/hyperactivity disorder 4, and the complex pathophysiology of Parkinson's syndrome 5, where altered norepinephrine signaling affects both motor and non-motor symptoms. In the case of Parkinson's disease (PD), NET's role extends beyond the central nervous system to implicate peripheral sympathetic denervation 6. Accumulation of alpha-synuclein, a hallmark of Parkinson's pathology 7, in the peripheral autonomic nervous system may lead to impairment of NET function 8. This phenomenon not only contributes to the diverse autonomic symptoms experienced by patients but also serves as a potential marker for early diagnosis 9. Furthermore, NET is a key player in the growth and progression of certain tumors 10, including neuroblastoma 11, pheochromocytoma 12, and paraganglioma 13. The expression of NET in these malignancies provides a unique target for both diagnostic imaging and radiotherapeutic interventions 14, allowing for the precise localization and treatment of these tumors. As we advance our understanding of NET's roles in these varied conditions, the development of targeted radiopharmaceuticals presents an opportunity to leverage this transporter for improved diagnostic accuracy and the delivery of targeted therapies, ultimately aiming to enhance patient outcomes in a multitude of clinical settings.

**Figure 1:**
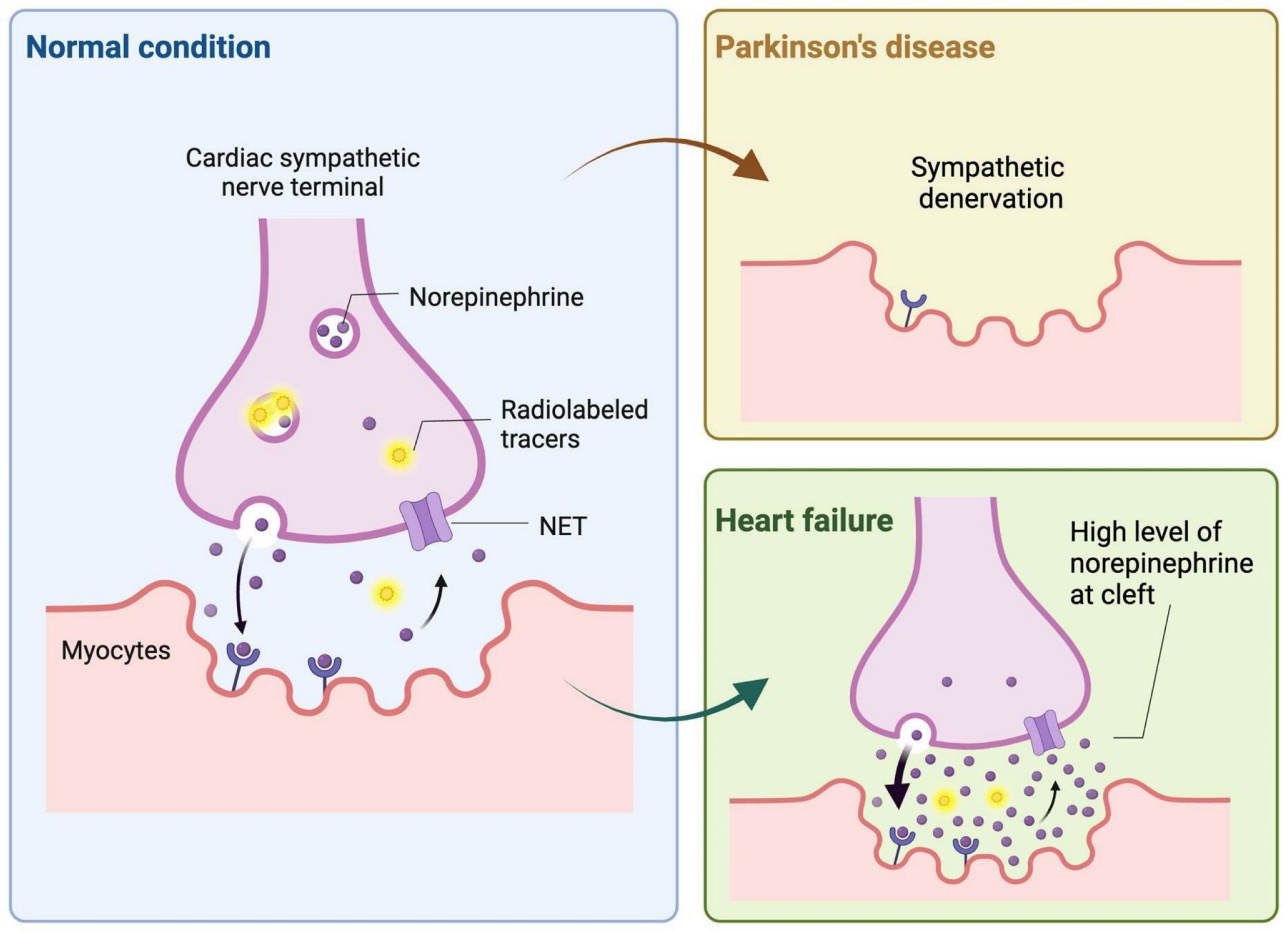
Illustration of the dynamics of norepinephrine and analogous radioactive tracers at cardiac sympathetic nerve endings. It depicts how Parkinson's disease and heart failure affect cardiac tracer uptake. In Parkinson's disease, the accumulation of alpha-synuclein at the terminals of cardiac sympathetic nerves leads to their denervation, resulting in reduced tracer uptake by the heart. Conversely, in heart failure, there is an upregulation of sympathetic nervous activity, which elevates norepinephrine levels in the synaptic cleft, thereby inhibiting tracer uptake due to competitive mechanisms. Created with BioRender.com.

*Introduction to radiopharmaceuticals and their diagnostic/therapeutic uses*. Radiopharmaceuticals represent a unique intersection of pharmacy and nuclear medicine, serving as pivotal tools for both diagnosis and therapy in a myriad of diseases 15. These specialized compounds are composed of a radioactive isotope, known as a radionuclide, which is bound to a pharmaceutical agent. The agent acts as a carrier, delivering the radionuclide to specific cells, tissues, or organs, while the emitted radiation allows for imaging or therapeutic action. In diagnostics, radiopharmaceuticals are utilized primarily for their ability to emit gamma rays, which can be detected by imaging techniques such as Single Photon Emission Computed Tomography (SPECT) 16. These modalities offer a non-invasive means to visualize biological processes in vivo. For example, ^123^I-labeled meta-iodobenzylguanidine (MIBG) is specifically taken up by neuroendocrine cells via NET, enabling the imaging of diseases like pheochromocytoma and neuroblastoma 17, as well as providing cardiac sympathetic innervation insights in heart failure patients 18 (**Figure 1**). Therapeutically, radiopharmaceuticals exploit the cytotoxic effects of radiation, targeting and damaging diseased cells while sparing healthy ones to a certain extent. Beta-emitters like ^131^I, used in MIBG therapy, have been employed to deliver targeted radiotherapy to neuroendocrine tumor cells, which take up the MIBG compound, allowing the attached ^131^I to irradiate and kill the tumor from within 19, 20. Over the past decade, the field of radiopharmaceuticals is progressively advancing towards 'theranostics' — a therapeutic strategy that combines specific targeted therapy based on specific targeted diagnostic tests, i.e. it combines therapeutic and diagnostic capabilities into a single agent or closely related agents. This approach ensures that the patients who are most likely to benefit from a particular treatment are identified using a corresponding diagnostic radiopharmaceutical 21.

*Current challenges in NET targeting precision medicine.* Precision medicine targeting NET faces several challenges that underscore the need for advancements in both diagnostic and therapeutic radiopharmaceuticals. One significant hurdle is the limited resolution of ^123^I-labeled MIBG imaging with SPECT. While SPECT is a mainstay of clinical nuclear medicine in the diagnosis of diseases like neuroblastoma 22, its dependency on lead collimators and the inherent physical limitations of gamma camera design result in less-than-optimal spatial resolution. This is particularly problematic in pediatric patients with neuroblastoma, where the small body size necessitates imaging that can discern fine anatomical details to accurately localize disease and guide treatment. Furthermore, the current gold-standard therapeutic radiopharmaceutical, ^131^I-labeled MIBG, demonstrates limited effectiveness in some clinical settings exemplified by a 39% objective response rate in neuroblastoma patients 23. Despite being one of the most effective treatments for neuroblastoma, mortality rates remain concerning, with a significant proportion of patients not achieving long-term survival post-treatment 24. This reality brings to the forefront an urgent need for the next generation of radiopharmaceuticals that can deliver more precise imaging and potent therapeutic effects. These challenges not only call for improved spatial resolution in diagnostic imaging to facilitate early and accurate detection, especially in smaller lesions, but also for the development of more effective therapeutic agents that can translate into improved survival rates. The next generation of precision medicine aims to address these gaps by allowing for tailored diagnosis and treatment based on individual patient characteristics, harnessing the power of advanced radiopharmaceuticals to enhance patient care and outcomes.

## The Evolution of Diagnostic Radiopharmaceuticals

*History of SPECT and PET imaging.* The evolution of SPECT and Positron Emission Tomography (PET) imaging has dramatically advanced nuclear medicine, each offering distinct capabilities based on their underlying technology. SPECT imaging, which matured as a clinical tool in the 1970s, relies on gamma cameras equipped with lead collimators to detect gamma rays emitted from radiopharmaceuticals 25. These collimators, necessary to direct the photons onto the detector, are a fundamental component of SPECT technology. However, they inherently limit the system's spatial resolution due to the trade-off between sensitivity and resolution 26. In practice, this results in SPECT practical resolutions often being larger than 10mm, which can be insufficient for detecting small lesions, a limitation especially critical in pediatric oncology.

PET technology, which became widely available in the 1980s 27, marked a significant improvement over SPECT in terms of both sensitivity and resolution 28. In contrast, PET's ability to detect coincident photon pairs from positron annihilation events eliminates the need for lead collimators. This increases PET's sensitivity and allows for more precise spatial resolution, typically around 4-5mm 29. The high sensitivity of PET also enables dynamic imaging 30, providing real-time tracking of radiotracer kinetics for comprehensive functional assessments 31. The introduction of whole-body PET imaging has been revolutionary, especially in oncology, where it has become a cornerstone for accurate tumor staging and therapy monitoring 32. PET's intrinsic technical advantages have made it a preferred modality in many clinical scenarios, leading to an increase in its adoption over SPECT. This shift is driven by PET's comprehensive diagnostic capabilities, which support a more personalized approach to patient care. The limitations of SPECT, including its reliance on lead collimators and lower spatial resolution, have prompted ongoing efforts to enhance its performance. Meanwhile, PET continues to advance with the development of new class of radiotracers and the integration of multimodal imaging systems such as PET/MRI 33. The digitalization of detectors 34, improvement in scintillator materials 35, and refinements in image reconstruction algorithms 36 have all contributed to the superior performance of PET. Moreover, the introduction of time-of-flight (TOF) has opened new avenues for more detailed and informative imaging 37.

*^123^I-MIBG in Neuroendocrine Imaging.* ^123^I-MIBG, currently the primary clinically-used NET-targeting radiotracer, utilizes SPECT technology to diagnose cardiac diseases and provide independent risk stratification through adrenergic imaging 38. As a pioneer in the benzylguanidine series, ^123^I-MIBG's polar structure allows for long-term storage in vesicles post-uptake, and its stability against monoamine oxidases breakdown exemplifies its well-designed nature 39. The usefulness of ^123^I-MIBG has been substantiated by substantial clinical trial data, notably from such as ADMIRE-HF study 40. Its application extends to risk stratification for the potential implantation of cardioverter defibrillators (ICD) in heart failure patients, with the ongoing ADMIRE-ICD trial set to further elucidate its impact on patient management 41. For endocrine-related malignancies with high NET expression, ^131^I-MIBG serves as a therapeutic agent against in certain adult and pediatric patients with pheochromocytoma, paraganglioma and neuroblastoma 19, 42, 43, taking advantage of the cells' uptake and storage of monoamines. Beyond above applications, the utility of ^123^I-MIBG and related NET radiotracers is expanding into other pathophysiological assessments involving peripheral sympathetic activity, such as Parkinson Disease (PD) 44 (**Figure 1**). The presence of alpha-synuclein pathology in the peripheral nerve system is a characteristic feature of PD 45, 46, and utilizing these radiotracers aids in distinguishing PD from other neurodegenerative diseases. Furthermore, this diagnostic approach highlights the systemic nature of PD, extending its effects beyond the central nervous system 47. Recent studies suggest that ^123^I-MIBG scintigraphy, often used alongside ^123^I-ioflupane SPECT, provides substantial diagnostic accuracy in differentiating PD from atypical parkinsonism and may contribute to the identification of early-stage PD 48. Additionally, ^123^I-MIBG is proving valuable in distinguishing Alzheimer's disease from Lewy Body dementias 49. Dynamic SPECT imaging using ^123^I-MIBG has also been applied to refine the quantification of cardiac nerve activity 50. The process involved multiple scans and blood analyses for radiometabolites and a population-based correction method was then applied to the dataset, which significantly improved the measurement of the drug's distribution in heart tissue compared to previous methods. This advancement promises to enhance the precision of cardiac nerve function evaluations in medical practice.

**Figure 2:**
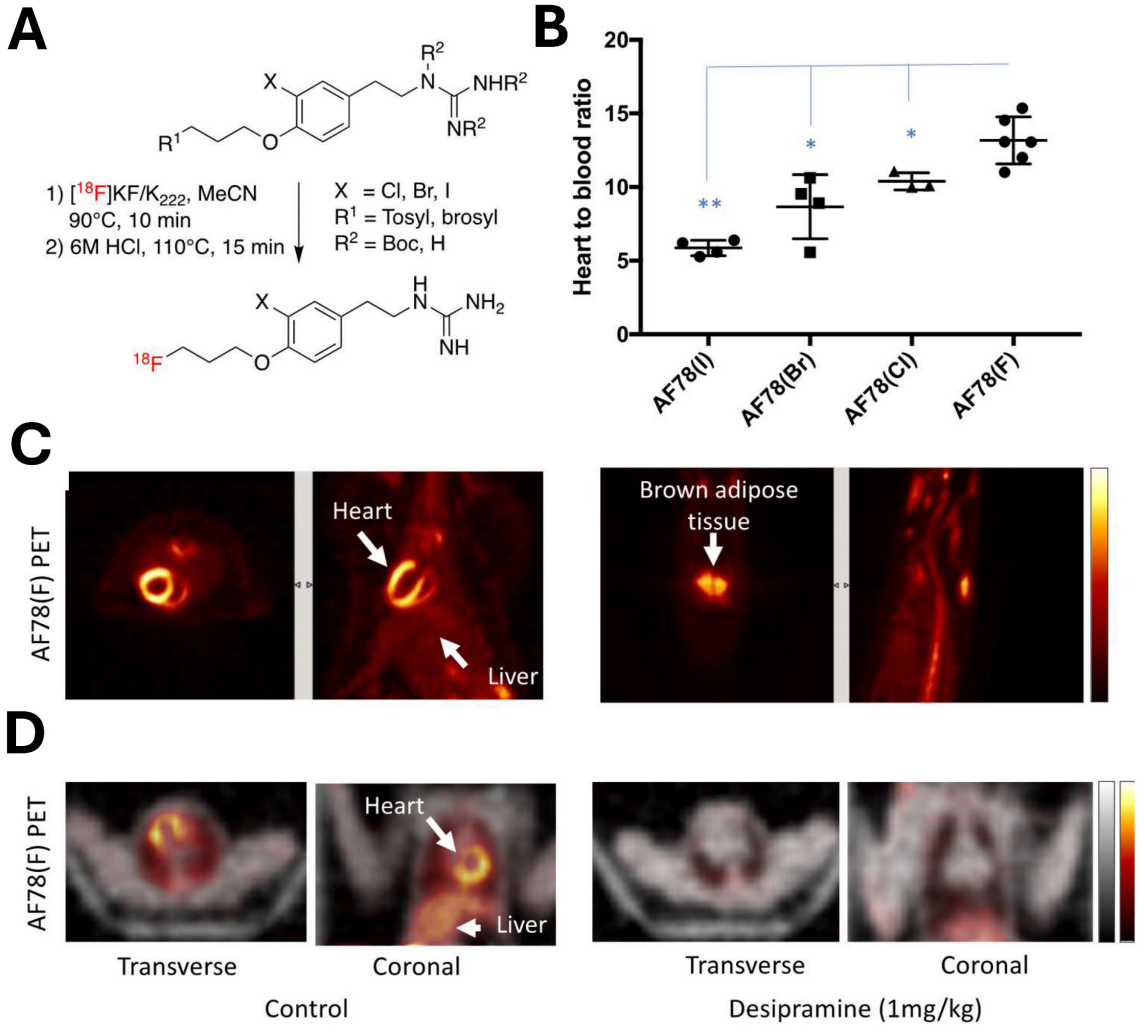
^18^F-AF78 demonstrated high NET affinity and advantageous in vivo radiotracer kinetics across various species. (A) A protocol for the one-pot, two-step radiolabeling of NET-targeting tracer ^18^F-AF78. (B) In vivo studies of radiotracer distribution in healthy rats confirm that ^18^F-AF78(F) showing highest contrast cardiac uptake among meta-substituents on the benzene ring. (C) PET scans reveal clear imaging of cardiac tissue (left) and brown adipose tissue uptake (right) in healthy rats using ^18^F-AF78(F). (D) Comparative PET scans of non-human primates (NHPs), with and without the NET-selective inhibitor desipramine, demonstrate the specificity of ^18^F-AF78 in cardiac imaging. Adapted with permission from 73, Copyright 2023, MDPI.

**Figure 3:**
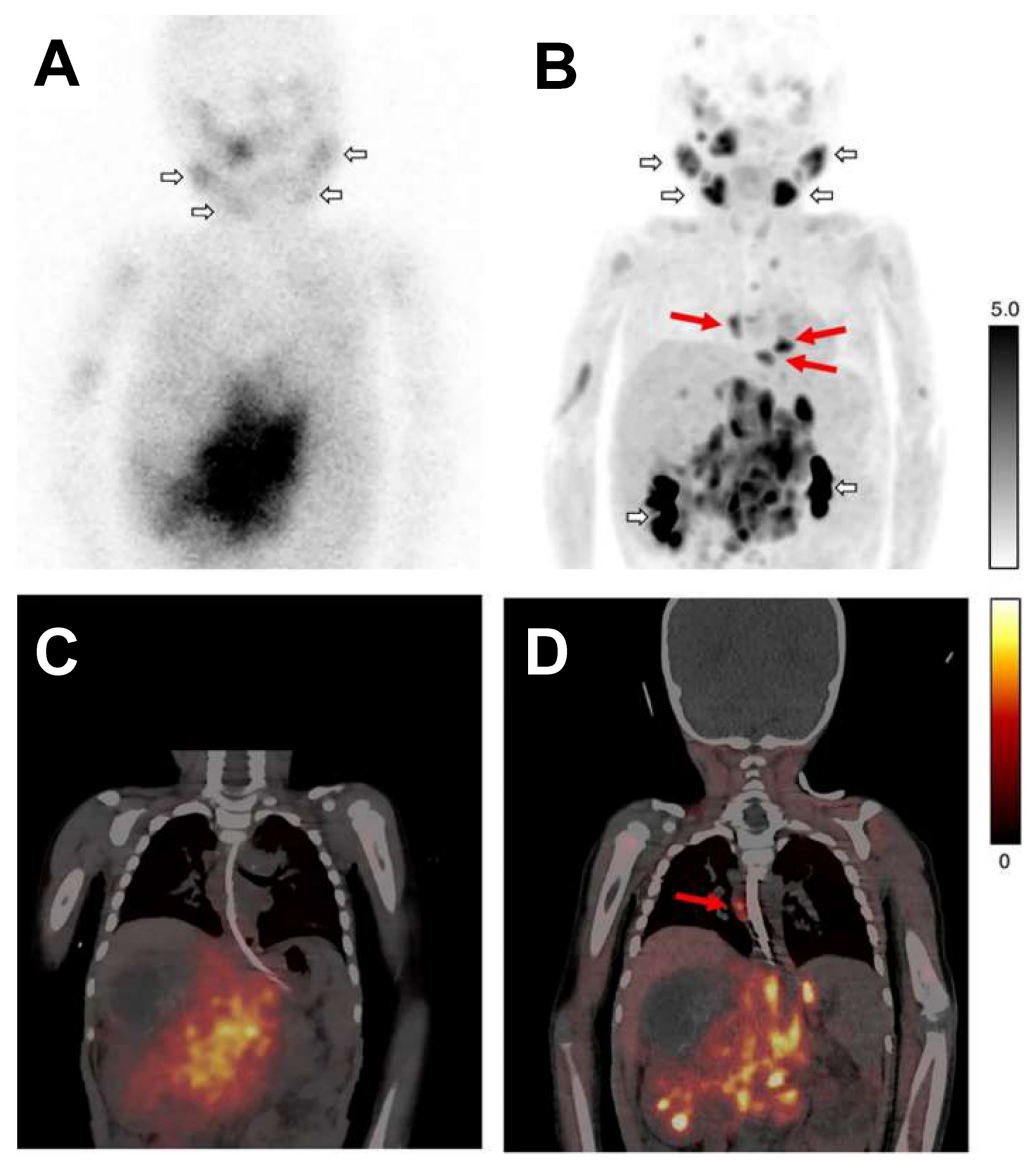
Planar scintigraphy with ^123^I-MIBG (A) and the corresponding fused SPECT/CT (B), along with ^18^F-MFBG PET maximum intensity projection (MIP) (C) and fused PET/CT (D), were conducted on a patient with neuroblastoma. Short arrows in images (A and C) illustrate normal physiological uptake in the salivary glands and the renal pelvicalyceal system. Areas of additional uptake represent tumor lesions. Long red arrows identify extra mediastinal lymph node metastases that are captured by ^18^F-MFBG PET, showcasing its superior sensitivity, but these were not on ^123^I-MIBG scans. Adapted with permission from 67 Copyright 2022, Springer Nature.

**Figure 4:**
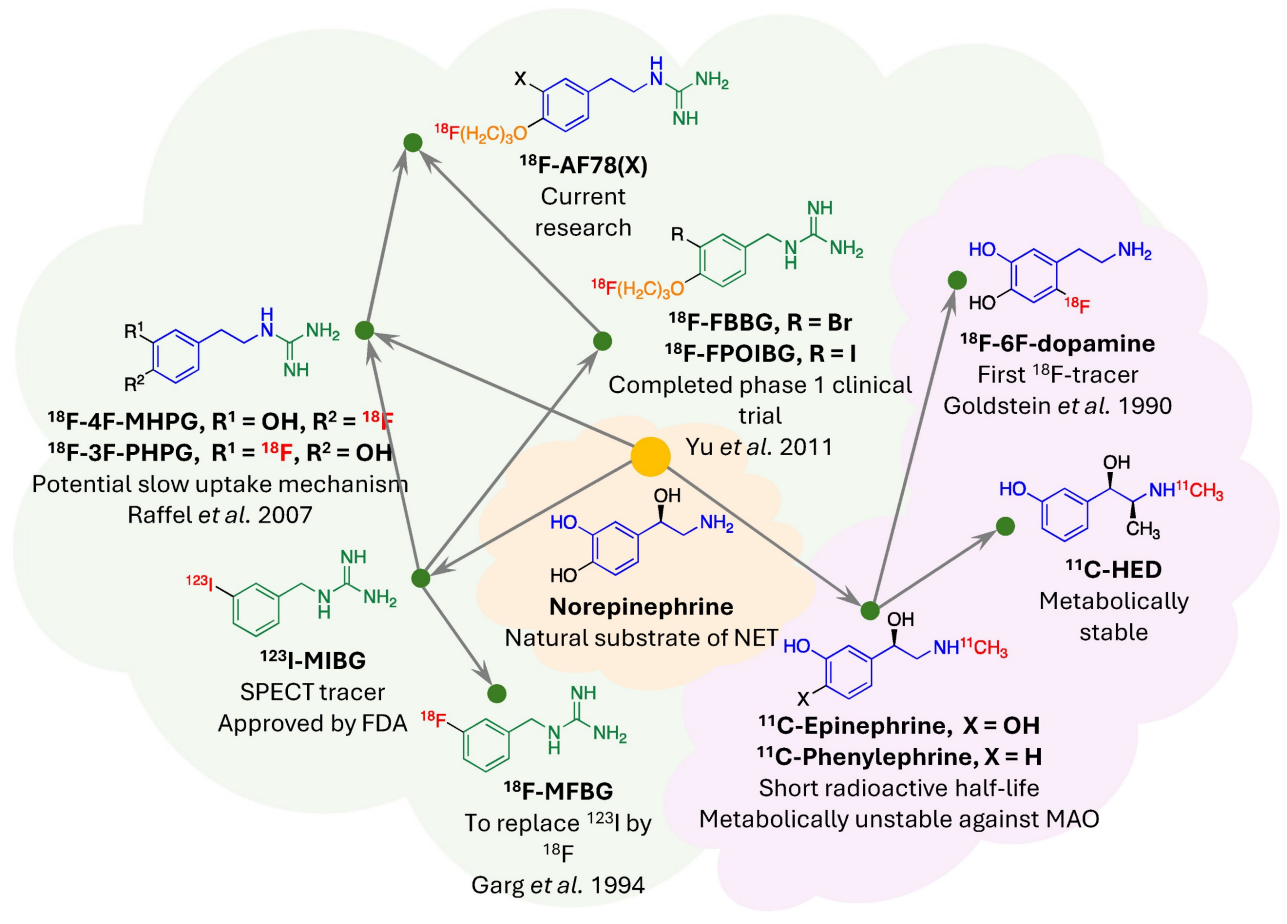
Evolutionary phylogeny of Norepinephrine Transporter (NET) radiotracers. This phylogenetic tree traces the development of selected NET-targeting radiotracers, classified into two main categories: monoamines (indicated by the violet cloud) and guanidines (denoted by the light green cloud). Shared molecular frameworks within the radiotracers are color-coded to illustrate structural similarities—core structures in blue and green, and the ubiquitous "tail" motif in orange. The placement of radionuclides within the molecular structures is highlighted in red. Adapted with permission from 73, Copyright 2023, MDPI.

*NET targeting PET tracers.* Despite its three-decade history, SPECT radiotracers like ^123^I-MIBG face limitations, such as inferior spatial resolution relative to PET, suboptimal sensitivity for small lesions or tumors, complex scanning protocols, and delayed imaging results. These drawbacks have spurred a shift in research focus towards developing PET tracers, which promise enhanced resolution and expedited diagnostic processes. ^18^F-labeled tracers, such as ^18^F-fluorodeoxyglucose (FDG), have revolutionized oncological imaging by enabling detailed whole-body scans that are critical for staging and assessing treatment response 51. The development of ^18^F-labeled tracers extends beyond FDG to include specific molecules targeting various biological pathways 52. These tracers are designed to bind to particular proteins or receptors, providing insights into a range of physiological and pathological processes. For instance, ^18^F-labeled compounds engineered to target NET offer enhanced precision in visualizing neurodegenerative diseases and sympathetically mediated cardiac conditions compared to the existing ^123^I-MIBG. The landscape of PET imaging has greatly evolved through the ongoing quest to enhance tracer specificity and NET binding affinity, key factors for precise diagnostic imaging 39. The narrative began with the advent of ^11^C-labeled tracers such as ^11^C-hydroxyephedrine (HED), an innovative tracer for delineating cardiac sympathetic nerve function 53-56. Although ^11^C-HED demonstrates high affinity for norepinephrine transport system and has been instrumental in cardiac 57 and oncological imaging 58, its clinical utility is curtailed by the short half-life of ^11^C (20.4 min), which requires the proximity of a cyclotron and constrains the imaging time window. Despite the achievements with ^11^C-labeled tracers, the shift toward ^18^F-labeling over the past two decades is driven by several advantages: cost-effectiveness due to centralized distribution, the feasibility of delayed and prolonged scans owing to a longer half-life (110 min), enhanced avaliablity of PET imaging, and the design flexibility that accommodates improved metabolic stability. These benefits are steering the development of new NET tracers towards ^18^F to leverage the full potential of PET imaging technology. With the unveiling of ^18^F-6F-dopamine 59, 60, ^18^F-LMI1195 61-66 and ^18^F-meta-fluorobenzylguanidine (MFBG) 67-69, the field witnessed enhanced tracers offering superior cardiac imaging contrast, thereby improving the delineation of sympathetic nervous activity within the heart 69, 70. This was a significant step forward in the cardiac applications of PET imaging, building on the foundations laid by its predecessors. The introduction of ^18^F-AF78 71-73 and ^18^F-PHPG/MHPG 74, 75 is a testament to the rapid progress in this domain, delivering tracers with pronounced NET affinity 72, potentially reshaping the diagnostic landscape for a variety of NET-related disorders. The high binding affinity of ^18^F-AF78 (**Figure 2**) 73 could lead to superior sensitivity and specificity in disease detection, especially in conditions where NET dysfunction is implicated, including certain neurodegenerative pathologies and neuroendocrine tumors. The evolution of these PET tracers showcases a decisive march toward imaging modalities that are not only more efficient but also molecularly astute, aligning with the overarching objectives of precision medicine. As we anticipate the future, the progression of NET-targeted PET tracers carries the potential to not only refine diagnostic accuracy (**Figure 3**) 67, 68 but also guide therapeutic interventions tailored to the unique molecular profiles of individual patients, truly embodying the essence of personalized care. An overview of the radiophamaceuticals targeting NET is shown in **Table 1** and **Figure 4**.

^124^I-labeled MIBG offers a promising PET/CT imaging alternative to the current SPECT/CT radiotracer ^123^I-MIBG, despite some inherent challenges. Research by Seo *et al.* has shown the potential of ^124^I-MIBG PET/CT in streamlining pretherapy dosimetry for ^131^I-MIBG therapy using just two time-point scans, providing an accurate estimation of tumor and organ doses 76. Aboian *et al.* further demonstrated ^124^I-MIBG PET/CT's superior lesion detection in children with neuroblastoma, identifying significantly more lesions than ^123^I-MIBG SPECT/CT 77. Furthermore, Maric *et al.* have successfully utilized dosimetry derived from ^124^I-MIBG PET scans to guide dosing in high-activity ^131^I-MIBG therapy, resulting in durable disease control with manageable side effects 78. These findings underline the diagnostic and therapeutic advantages of ^124^I-MIBG PET/CT, suggesting its role in enhancing the precision of metastatic neuroblastoma treatment. However, the broader clinical use of ^124^I is limited due to its high production costs and technical constraints 79. The isotope is produced via the ^124^Te(p,n)^124^I nuclear reaction in a cyclotron and has a half-life of 4.2 days, favorable for long-term biological studies. Yet, the high positron energy of ^124^I, at 2.14 MeV, leads to a wide positron range, potentially reducing PET imaging resolution due to the long travel distance of positrons before annihilation 79, 80. Nevertheless, the benefits of ^124^I, such as its longer half-life and robust chemistry that allows for versatile compound labeling, continue to drive interest in its use for medical imaging and therapeutic applications.

In a recent comparative study, ^18^F-MFBG PET/CT was shown to outperform ^123^I-MIBG SPECT/CT in detecting neuroblastoma lesions in children 68. The research included 40 patients, averaging 6 years old. It revealed that while both imaging modalities had negative results in six patients, ^18^F-MFBG PET/CT detected lesions in 11.8% of patients who were negative on ^18^F-MFBG. Overall, ^18^F-MFBG PET/CT identified 252 more lesions than ^123^I-MIBG SPECT/CT, marking a significant increase in detection. Additionally, Curie scores, which quantify tumor load, were considerably higher with ^18^F-MFBG PET/CT PET/CT, with 88.2% of patients showing active disease having higher scores compared to those from ^123^I-MIBG imaging. This suggests that ^18^F-MFBG PET/CT could offer improved diagnostic accuracy in pediatric neuroblastoma.

As the field of NET-targeting PET tracers continues to expand, we recognize that there are significant knowledge gaps and clinical challenges that must be addressed 39. A primary recommendation is to create a comprehensive database to systematically track and analyze the performance of ^18^F-labeled tracers across various clinical scenarios. This would not only help in recognizing patterns of efficacy and patient outcomes but also contribute to refining clinical guidelines and protocols. Further investigation comparing the biodistribution, such as liver and kidney uptake, and clearance rates across diverse patient cohorts would be beneficial, particularly when different tracers are used. This research is critical for optimizing both the performance of imaging and the dosing regimens, which in turn, can enhance the safety profile of these diagnostic agents. A lack of longitudinal data linking PET imaging results with long-term patient outcomes presents another gap. Consequently, there is a need for extended follow-up in multi-institutional studies to elucidate the prognostic value of these tracers in cardiology as well as in oncology, particularly in relation to therapeutic decisions and patient survival. Clinically, the underutilization of advanced NET-targeting tracers due to logistical barriers is a pressing issue. By forging partnerships between academia, industry, and healthcare systems, we can enhance the availability and application of these tracers, ensuring equitable access to this crucial diagnostic technology. Moreover, yet in the early development stage, with tracers such as ^18^F-AF78 72, 73 and ^18^F-PHPG/MHPG 75 demonstrating high NET affinity, refining imaging protocols becomes possible, which may significantly improve disease detection and characterization. By integrating these imaging results with molecular and genetic data, we could potentially further tailor the diagnosis and treatment of NET-related disorders, moving closer to the personalized medicine paradigm.

## NET-Targeted Therapy with Beta and Alpha Emitters

*^131^I-MIBG therapy's mechanisms and applications.* ^131^I-MIBG therapy, which harnesses the therapeutic properties of a radioactive isotope for the targeted treatment of certain neuroendocrine tumors, has seen a reaffirmation of its clinical value through the FDA approval in July 2018. ^131^I-MIBG functions on the principle of molecular mimicry. Structurally analogous to norepinephrine, it is actively transported into neuroendocrine cells via the NET. Once internalized, the radioactive decay of ^131^I releases high energy beta-particles, leading to localized cell damage and apoptosis, primarily within neuroendocrine tumors that overexpress NET. It is now an FDA-approved therapy for adult and pediatric patients (12 years and older) with locally advanced or metastatic pheochromocytoma or paraganglioma who require systemic anticancer therapy. This regulatory milestone has cemented ^131^I-MIBG's role in the treatment paradigm of these conditions. The implementation of ^131^I-MIBG therapy post-FDA approval requires multidisciplinary coordination to ensure patient safety and treatment efficacy 24. The procedure necessitates isolation due to the radioactive nature of the compound and involves specialized nuclear medicine facilities. Looking forward, optimizing dosing, mitigating side effects, and integrating ^131^I-MIBG with other therapies 81 remain areas of active research. ^131^I-MIBG therapy would provide a much-needed option for children with high-risk neuroblastoma, who may have limited treatment choices, and has been associated with improved survival outcomes in this patient population 81. As clinical experience with ^131^I-MIBG grows and further studies refine its application, it is poised to improve outcomes for patients facing limited treatment options, symbolizing progress in personalized cancer therapy.

Dosimetry in radionuclide therapy is the process of measuring and assessing the radiation dose that a patient receives during treatment with radioactive pharmaceuticals such as ^131^I-MIBG 82. The process begins with an initial tracer dose of the radiopharmaceutical to understand its distribution and clearance within the body, often involving several imaging sessions over time. From this, specialists calculate the radiation absorbed by different tissues, striving to maximize the dose to the tumor while limiting exposure to healthy organs to reduce toxicity. For ^131^I-MIBG therapy specifically, these dosimetry calculations are critical in shaping a personalized treatment plan. The therapeutic activity is carefully determined based on the patient's unique physiological and disease characteristics to deliver a defined whole-body dose aimed at achieving the best therapeutic effect. Advancements in dosimetry techniques have evolved to include SPECT-based tumor dosimetry, with studies showing the ability to deliver whole-body doses, fine-tuning the activity administered based on whole-body retention from the initial therapy 83. In 2016, a more individualized approach to ^131^I-MIBG therapy considered both dosimetry data and clinical judgment to deliver high activities of ^131^I-MIBG that patients could tolerate, especially with stem cell support 84. This personalized method demonstrated high response rates and manageable toxicity. Comparatively, low-dose diagnostic scans with ^123^I-MIBG were found to be less effective than post-treatment scans with ^131^I-MIBG in detecting malignant pheochromocytoma and paraganglioma, underscoring the importance of scan timing and dose in diagnostic accuracy 85.

Edmondson *et al.* explored gene expression analysis as an alternative biodosimetric approach to calculate internal radiation doses for neuroblastoma patients treated with ^131^I-MIBG 86. Blood samples from 40 patients, with a median age of 9, were collected before and 72 to 96 hours post-infusion. Absorbed doses were calculated using patient-specific data and compared with gene expression changes. Six genes were significantly modulated by the ^131^I-MIBG treatment. A predictive model using three gene transcripts could account for 98% of the variance in gene expression changes post-treatment, suggesting that these biomarkers are indicative of internal radiation exposure. This novel approach holds promise for biodosimetry in radiopharmaceutical therapy.

The treatment with ^131^I-MIBG can lead to certain side effects such as bone marrow suppression, thyroid gland damage, nausea, hypertension, and inflammation of the salivary glands 82, 87. Protective measures like thyroid-blocking agents, anti-emetics, blood pressure control, and hydration are standard to alleviate these effects. The integration of ^131^I-MIBG with ^90^Y-labeled DOTA-D-Phe1-Tyr3-octreotide (^90^Y-DOTATOC) has been proposed as a synergistic approach to enhance tumor dose delivery in neuroendocrine tumors while potentially reducing side effects 14. The theoretical model developed by Madsen *et al.* suggests that this combined therapy could significantly increase tumor dosage without surpassing critical organ dose limits, as these agents impact different dose-limiting tissues 88. Further research by Bushnell *et al.* through a phase 1 clinical trial has affirmed the practicality of this approach for patients with nonoperable progressive neuroendocrine tumors 89. In this trial, the combination of ^90^Y-DOTATOC with ^131^I-MIBG was adjusted based on subject-specific dosimetry with respect to renal and bone marrow dose constraints. The preliminary results showed that the combined therapy could achieve a tumor dose increment between 34-83% compared to ^90^Y-DOTATOC PRRT alone, without encountering dose-limiting toxicities and adhering to a dose limit of 1,900 cGy for kidneys and 150 cGy for bone marrow. These studies pave the way for more personalized and potentially more effective radionuclide therapy regimens for treating neuroendocrine tumors, underlining the necessity of comprehensive dosimetric analysis to fully harness the benefits of combined-agent therapy.

*Introduction of alpha-particle therapy.* Among various alpha-emitting radionuclides that are gaining interest 90, 91, astatine-211 (^211^At) 92 stands out for its potential to advance targeted cancer therapies, following the path laid by the halogen ^131^I 93. Both ^211^At and ^131^I are adept at creating stable, target-specific radiopharmaceuticals, especially for targeting the NET. ^211^At is particularly advantageous due to its emission of High-Linear Energy Transfer (LET) irradiation alpha particles 94, which confer a concentrated therapeutic effect. While the beta-particle emission of ^131^I-MIBG has established its role in treating neuroendocrine tumors, ^211^At-astatobenzylguanidine (^211^At-MABG), a structural analogue of MIBG, promises enhanced precision and cytotoxicity at the tumor site due to the localized impact of alpha particles. In addition, actinium-225 (^225^Ac) is also highly valued, particularly for its use in peptide-based tracers or antibody radioconjugates, due to its affinity for larger, metal-affinitive biological molecules and the possibility to be chelated by several commonly used macrocyclic chelators such as DOTA, making it a versatile choice for targeted therapy 95. In contrast, ^211^At is more compatible with small organic molecules to form stable covalent bond exemplified by MABG, broadening its applicability for various radiopharmaceuticals 96.

Both ^211^At and ^225^Ac show promise as alpha-emitting agents for medical purposes, yet they are markedly different in their production and decay characteristics. The generation of ^211^At generally requires a cyclotron 97, whereas ^225^Ac is typically sourced from nuclear reactors, with its longer half-life of 10 days enhancing its potential for wider distribution 98. Establishing in-house production of ^211^At using a medium-energy cyclotron signifies a significant capital investment for medical or research institutions. This outlay encompasses not only the purchase price but also the subsequent costs related to maintenance, operations, and the skilled personnel necessary to ensure safe and efficient facility operation. Given the short half-life of ^211^At — a mere 7.2 hours in contrast to the 10-day half-life of ^225^Ac — there are critical differences impacting both the biodistribution for effective dose delivery and logistical considerations 99. The relatively short half-life of ^211^At necessitates a production site close to the clinical application venue or an expedient transport system to deliver the isotope promptly and with minimal loss of activity. Such logistics require a synchronized supply chain to guarantee consistent availability.

A significant advantage of ^211^At come from its non-serial decay property. It transitions directly to stable Bismuth-207 (^207^Bi), in contrast to ^225^Ac, which initiates a decay chain yielding multiple radioactive daughters 95. The immediate transition to a stable state for ^211^At means there is no additional radiation emitted following its decay, streamlining many facets of its therapeutic application and related safety measures. In clinical practice, managing ^211^At focuses on the direct administration and adherence to safety protocols until it decays to its stable form. This is unlike the approach required for serially decaying nuclides like ^225^Ac, which might need consideration for the management of radioactive decay products over a more extended period. These production nuances, along with subsequent purification, logistical arrangements, and regulatory considerations, highlight the unique challenges and potential of each radionuclide within precision medicine's evolving landscape.

**Figure 5:**
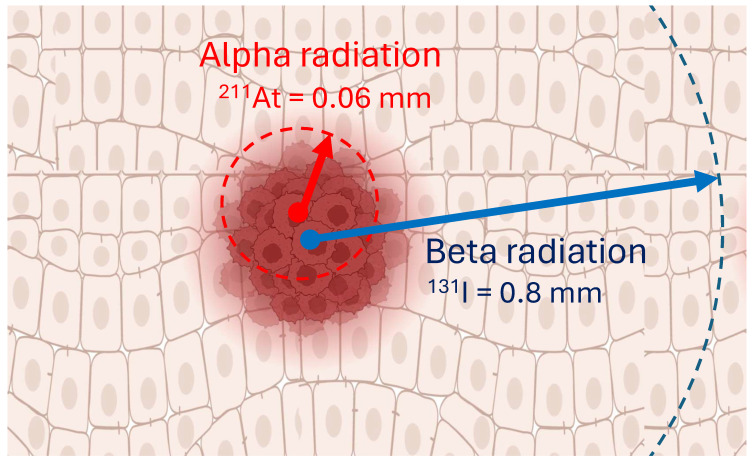
Alpha-particles, like ^211^At, offer the potential to combine cell-specific molecular targets with radiation that has a range in tissue of only a few cells (0.06 mm), which is different to beta-particles, such as ^131^I, that have a maximum range of around 0.8 mm. The distinct advantages of alpha particles, such as their potent cytotoxicity and selective targeting, position targeted alpha therapy as a promising avenue in the field of cancer treatment. Created with BioRender.com.

**Figure 6:**
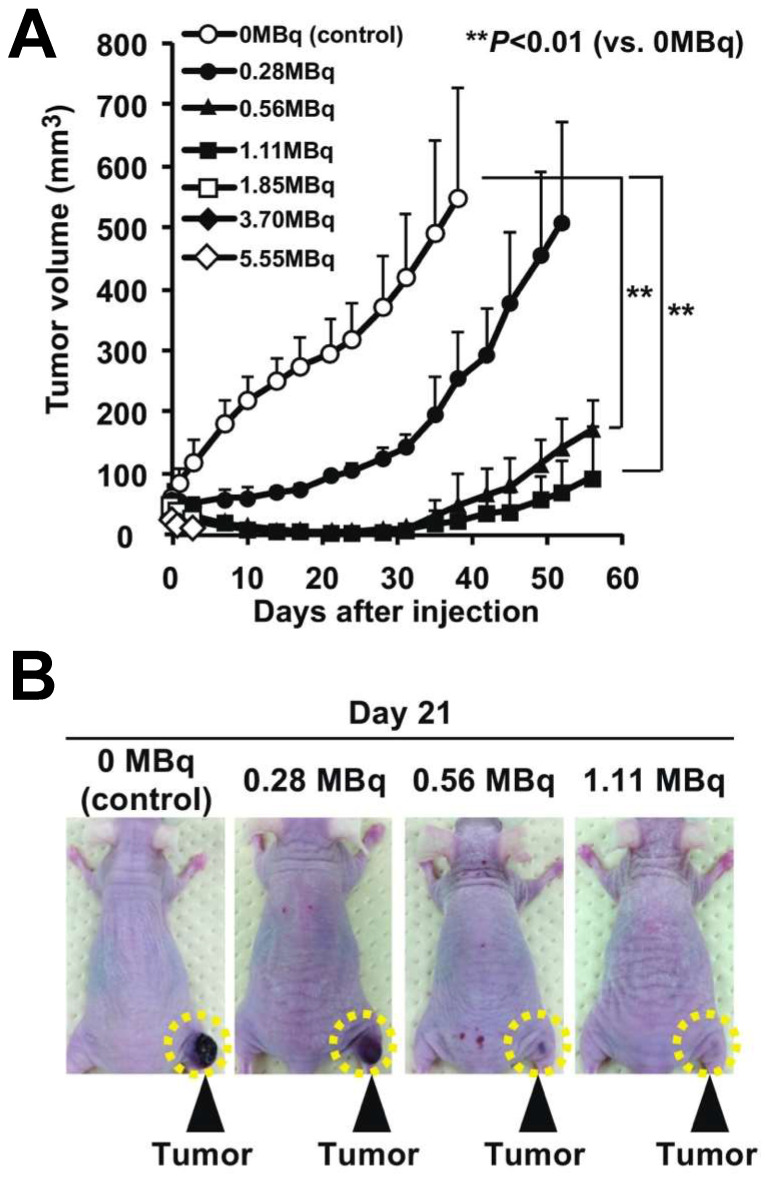
The ^211^At-MABG-treated mice showed significantly lower relative tumor growth in mice with PC12 pheochromocytoma cells: a) Tumor growth trajectories following ^211^At-MABG administration demonstrate significant reduction. b) Day 21 post-treatment images of mice, comparing ^211^At-MABG treatment, with tumors highlighted by dashed circles. Adapted with permission from 100. Copyright 2018, Springer Nature.

^211^At-MABG's superior localized effect is a consequence of the alpha-particles' short path length, resulting in intense cellular damage confined to a narrow range, thereby mitigating the risk to adjacent healthy tissue (**Figure 5**). This enhanced localized effect positions ^211^At as a formidable candidate for more focused and potent treatment modalities. Building upon iodine-based therapies, ^211^At-MABG has already demonstrated significant promise in preclinical experiments with neuroendocrine tumor models (**Figure 6**) 100, indicating potential for high efficacy and a favorable safety margin.

In Japan, a Phase 1 clinical trial (jRCT2021220012) is currently evaluating the pharmacokinetics, safety, and efficacy of ^211^At-MABG in adult patients with pheochromocytoma or paraganglioma who are not candidates for surgery or curative external radiation. This study utilizes a 3+3 dose-escalation design to determine the maximum tolerated dose, starting with three cohorts receiving increasing doses of the radiopharmaceutical (Cohort 1 = 0.65 MBq/kg, Cohort 2 = 1.3 MBq/kg and Cohort 3 = 2.1 MBq/kg). Key endpoints include dose-limiting toxicity, radioactive pharmacokinetics, urinary discharge of radioactivity, catecholamine response, objective response rate, progression-free survival, the impact of ^131^I-MIBG scintigraphy on tumor accumulation, and patient quality of life. As ^211^At-MABG is on early phase human trials, backed by encouraging animal study results, it stands on the cusp of transitioning from an experimental therapy to a tangible clinical reality. Research is now concentrated on refining ^211^At delivery and dosimetry accounting for its shorter physiological half-life compared to ^131^I 94, 101-104.

The therapeutic effects of ^211^At-MABG on malignant pheochromocytoma using RNA-sequencing to understand its molecular mechanisms and identify potential biomarkers for therapeutic response have also been explored 104, 105. In this regard, in vitro experiments on a rat pheochromocytoma cell line (PC12) were conducted, revealing significant anti-tumor effects. Transcriptomic analyses were performed at 3, 6, and 12 hours post-treatment with ^211^At-MABG, using iso-survival doses of 0.8 and 0.1 kBq/mL. Comparison with ^60^Co gamma-ray irradiation highlighted ^211^At-MABG-specific gene expression changes. Notably, four genes (Mien1, Otub1, Vdac1, and Vegfa) showed promise as potential therapy biomarkers due to their expression patterns correlating with survival decrease. This study's findings underscore the potential of ^211^At-MABG in treating malignant pheochromocytoma and pave the way for using molecular imaging to target identified biomarkers for enhanced therapeutic efficacy.

Enhanced imaging and computational modeling are needed to ensure accurate tumor targeting and minimized radiation exposure to non-targeted tissues, optimizing both therapeutic impact and patient safety 103. Clinical challenges also include establishing clear protocols for managing potential side effects unique to alpha-emitting therapies and ensuring access to these treatments across diverse healthcare settings. A roadmap for the field should involve the development of multicenter collaborations to share knowledge, standardize treatment protocols, and pool resources for large-scale trials that can provide the level of evidence needed for regulatory approval and clinical acceptance.

*PET diagnostics using ^18^F-tracers and ^211^At therapy can complement each other.* The synergy of NET-targeting PET diagnostics using ^18^F-labeled radiotracers together with ^211^At-MABG therapy embodies a promising approach to enhance the management of neuroendocrine tumors. The ^18^F-labeled NET-targeting PET tracers offer superior spatial resolution and the potential for precise quantification, which could significantly improve the identification and characterization of tumors with high NET expression 68, 106. This advanced diagnostic capability is expected to refine patient selection, incorporating potential biomarkers, specific patient characteristics, and disease stages, ensuring that the potent ^211^At-MABG therapy is administered to individuals most likely to benefit. ^211^At-MABG, targeting the NET for therapeutic delivery, leverages the cell-killing properties of alpha-particles 94, 99. This therapeutic strategy aims to maximize tumor eradication while limiting collateral damage to surrounding tissues due to the short range of alpha emission. When integrated with ^18^F-tracer PET imaging, clinicians could monitor therapeutic response, allowing for dynamic treatment adjustments.

**Figure 7:**
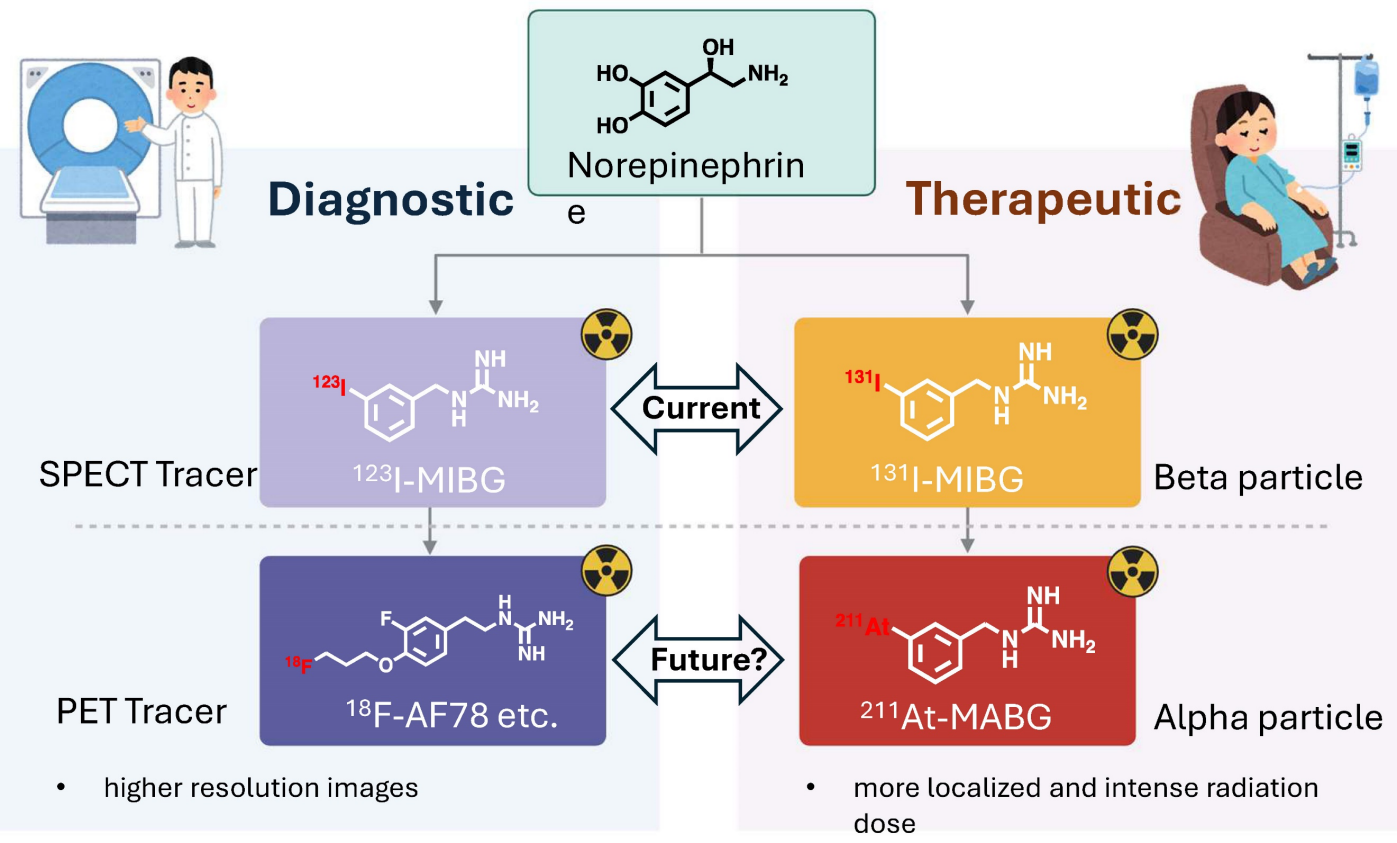
Advancements in Radiopharmaceuticals for NET Targeting: This illustration summarizes the progress in diagnostic and therapeutic agents, highlighting the integration of PET for high-resolution, sensitive imaging, and the employment of alpha-emitting nuclides for focused radiation therapy. These innovations herald a new era of precision medicine with enhanced diagnostic and treatment capabilities. Created with BioRender.com.

While this integrative model of ^18^F NET-targeting PET diagnostics and ^211^At-MABG therapy is intuitively appealing, it remains a hypothesis that demands empirical validation. It outlines the design of future studies that should aim to establish a correlation between ^18^F-labeled PET tracer uptake and NET expression with patient outcomes post ^211^At-MABG therapy to confirm the predictive value of PET imaging and to evaluate long-term outcomes and quality of life in patients undergoing this combined treatment approach, to ensure that the clinical benefits justify its use over conventional methods. In essence, there is a need for prospective clinical trials with clearly defined endpoints, encompassing both response criteria and patient-centric outcomes. Such trials would not only substantiate the therapeutic merits but also refine the selection criteria for patients most likely to benefit from this approach, thereby advancing the field towards more effective and individualized cancer therapies. If proven successful, this approach could significantly impact the trajectory of personalized cancer therapy, aligning treatment strategies closely with the unique biological signatures of each patient's tumor (**Figure 7**).

## Conclusion

The advent of ^18^F-labeled NET-targeting PET tracers and ^211^At therapy marks a transformative era in precision medicine. These novel approaches have opened up unprecedented avenues for managing neuroendocrine-related conditions. The sensitivity and specificity of ^18^F-labeled PET tracers for the NET allow for earlier and more accurate detection of diseases, thus streamlining the diagnosis and staging process for conditions such as heart failure, Parkinson's syndrome, and various neuroendocrine tumors. ^211^At therapy, leveraging targeted alpha-particle radiation, stands on the cusp of redefining the treatment landscape for NET-expressing tumors with its potential to deliver highly potent cytotoxic effects precisely where needed. The promise of these radiopharmaceuticals is predicated on the continuation of rigorous research to substantiate their therapeutic value and confirm their safety profile. As clinical trials progress, it is imperative that the medical community nurtures cross-disciplinary collaboration to enhance treatment methodologies, fully elucidate the long-term ramifications of these therapies, and innovate further in the development of even more targeted radiopharmaceuticals. Looking forward, NET-targeted radiopharmaceuticals are anticipated to significantly influence patient care. The integration of PET diagnostics using ^18^F-tracers, along with the therapeutic potential of ^211^At exemplified by MABG, could herald an era of highly individualized care, optimizing treatment plans to match the unique biological landscape of each patient. The potential of these modalities extends beyond immediate clinical outcomes, offering the possibility of not just improved management but also potentially increasing survival rates and the quality of life for those battling complex neuroendocrine conditions. As we stand at the threshold of this new chapter in medical science, NET-targeted radiopharmaceuticals offer a beacon of hope, illuminating the path toward a future where precision medicine is not just an aspiration but a reality.

## Figures and Tables

**Table 1:** Summary of NET targeting radiophamacuticals

Name	Development stage	Major characteristics	References
*Diagnostic*			
^123^I-MIBG	FDA approved for pheochromocytoma and neuroblastoma, Phase 3 completed (NCT02043522: heart failure)	SPECT tracer	38, 41, 44
^11^C-HED	Used in clinical trials, NCT00756366: heart failure, NCT 01400334: arrhythmic events)	PET tracer with short half-life	55, 56
^18^F-6F-dopamine	Investigational New Drug application Nr.138638: neuroblastoma	First ^18^F PET tracer for NET	59, 60
^18^F-MFBG	Phase 3 ongoing (NCT04724369: neuroblastoma) Clinical trial (NCT06149195: heart failure)	Structure comparable to MIBG	67, 69
^18^F-LMI1195	Phase 1 completed (NCT00891241, heart failure)	High radiochemical yield	70
^18^F-4F-PHPG	Early Phase 1 completed (NCT02385877: neuroendocrine tumors)	Slow NET kinetics and fast liver washout	74
^18^F-AF78	Preclinical stage (non-human primates)	High NET affinity in cellular assay	71-73
^124^I-MIBG	Phase 1 / 2 completed (NCT02043899: neuroblastoma)	PET tracer identical chemical property with ^123^I-MIBG	77, 78
*Therapeutic*			
^131^I-MIBG	FDA approved: pheochromocytoma and paraganglioma Phase 2 ongoing (NCT03561259: neuroblastoma)	Beta radionuclide therapy	19
^211^At-MABG	Phase 1 ongoing (JRCT2021220012: pheochromocytoma, paraganglioma)	Alpha radionuclide therapy	94, 101, 102
